# Cytoprotective Small Compound M109S Attenuated Retinal Ganglion Cell Degeneration Induced by Optic Nerve Crush in Mice

**DOI:** 10.3390/cells13110911

**Published:** 2024-05-25

**Authors:** Jonah J. Scott-McKean, Mieko Matsuyama, Charles W. Guo, Lin Ni, Brandon Sassouni, Shree Kurup, Robert Nickells, Shigemi Matsuyama

**Affiliations:** 1 Department of Ophthalmology and Visual Sciences, Case Western Reserve University, Cleveland, OH 44106, USA; jjs248@case.edu (J.J.S.-M.); charlesguo2345@gmail.com (C.W.G.); bxs598@case.edu (B.S.); shree.kurup@uhhospitals.org (S.K.); 2 Department of Ophthalmology and Visual Sciences, School of Medicine, University of Wisconsin (Madison), Madison, WI 53706, USA; nickells@wisc.edu; 3 Division of Hematology and Oncology, Departments of Medicine, Pharmacology and Pathology, School of Medicine, Case Western Reserve University, Cleveland, OH 44106, USA; 4 Case Comprehensive Cancer Center, Cleveland, OH 44106, USA

**Keywords:** apoptosis, BAX, cell death inhibitor, nerve injury

## Abstract

BAX plays an essential role in retinal ganglion cell (RGC) death induced by optic nerve injury. Recently, we developed M109S, an orally bioactive and cytoprotective small compound (CPSC) that inhibits BAX-mediated cell death. We examined whether M109S can protect RGC from optic nerve crush (ONC)-induced apoptosis. M109S was administered starting 5 h after ONC for 7 days. M109S was orally administered in two groups (5 mg/kg twice a day or 7.5 mg/kg once a day). The retina was stained with anti-BRN3A and cleaved Caspase-3 (active Caspase-3) that are the markers of RGC and apoptotic cells, respectively. ONC decreased the number of BRN3A-positive RGC and increased the number of active Caspase-3-expressing apoptotic cells. In ONC-treated retina, there were cells that were double stained with anti-BRN3A and ant-cleaved Caspase-3, indicating that apoptosis in BRN3A-positive RGCs occurred. M109S inhibited the decrease of BRN3A-positive cells whereas it inhibited the increase of active Caspase-3-positive cells in the retina of ONC-treated mice, suggesting that M109S inhibited apoptosis in RGCs. M109S did not induce detectable histological damage to the lungs or kidneys in mice, suggesting that M109S did not show toxicities in the lung or kidneys when the therapeutic dose was used. The present study suggests that M109S is effective in rescuing damaged RGCs. Since M109S is an orally bioactive small compound, M109S may become the basis for a portable patient-friendly medicine that can be used to prevent blindness by rescuing damaged optic nerve cells from death.

## 1. Introduction

Optic nerve injury, whether due to trauma, ischemia, or degenerative diseases, can result in severe vision impairment or even permanent blindness [1]. Optic nerve crush (ONC) is a commonly used animal model to study the effects of traumatic injury on the optic nerve [2]. ONC results in the physical disruption of axons and the surrounding tissues, leading to significant death of retinal ganglion cells (RGCs), the neurons that form the optic nerve [2]. 

BAX is a pro-apoptotic protein belonging to the *Bcl-2* family of proteins, which plays a critical role in regulating programmed cell death [3,4]. Previous studies demonstrated that BAX-mediated cell death plays an essential role in RGC degeneration induced by ONC [5,6,7]. Mechanistically, upon activation, BAX translocates to the mitochondria from the cytosol, where it promotes mitochondrial outer membrane permeabilization (MOMP) and the release of apoptotic factors, triggering the caspase cascade ultimately leading to cell death (Reviewed in [8]). For example, the mitochondrial translocation of BAX was observed in RGC after ONC [9,10], and ONC-induced RGC death was significantly attenuated in *BAX*-deficient mice [3]. In addition, RGC death caused by chronic elevation of intraocular pressure (IOP) was also attenuated by *BAX*-deficiency [10,11]. These results suggest that BAX is a potential therapeutic target to rescue RGC from optic nerve injury and other pathological conditions including chronic high levels of IOP.

Recently, we succeeded in developing cytoprotective small compounds (CPSCs) as potent BAX inhibitors [12]. M109S, one of CPSCs, is orally bioactive and penetrates the blood–brain/retina barriers [12]. In our previous study, M109S (oral gavage administration) protected retinal photoreceptor cells from light-induced cell death in mouse models of macular degeneration (*Abca4/Rdh8*-mutant BALB/c mice) [12]. Here, we report that oral administrations of M109S protected RGC from ONC-induced cell death.

## 2. Materials and Methods

### 2.1. Animals

Both male and female 6–8-week-old C57BL/6J mice (The Jackson Laboratory, Bar Harbor, ME, USA) were used in this study. The animals were housed in a temperature-controlled room with a 12-h light/dark cycle and free access to food and water or food restricted from 9:00 a.m. to 5:00 p.m. All animal experiments were approved by the Institutional Animal Care and Use Committee and were performed in accordance with the guidelines of the National Institutes of Health (project number 2020-0039; 2020).

### 2.2. ONC Procedure

All cohorts were subjected to a surgical procedure that involved crushing the optic nerve in one eye. A total of 8–10 mice were used in each cohort. The other eye was a control and underwent sham surgery without nerve damage. ONC was performed using previously described methods [13,14], which are briefly described here. The ONC procedure was as follows: The mice were anesthetized by an intraperitoneal injection of a ketamine and xylazine cocktail. Under a dissecting microscope, a lateral canthotomy was performed, and a small incision was made in the conjunctiva to access the optic nerve. The eye was gently retracted to expose the optic nerve, and the orbital muscles were deflected. The optic nerve, located 1–2 mm from the eyeball, was clamped for 5 s using self-closing N7 forceps (Fine Science Tools, Foster City, CA, USA). M109S or vehicle was orally administered for three days or one week after ONC surgery, and the mice were then euthanized, and their retinas were removed.

### 2.3. M109S Treatment 

**Figure 1:**
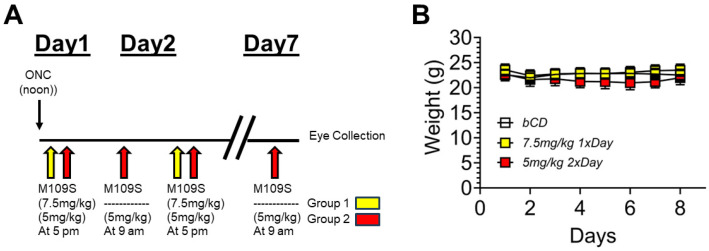
Experimental design of optic nerve crush (ONC) and M109S treatment. (**A**) Time schedule of the experiments. Group 1: M109S (7.5 mg/kg; Yellow) was administered at 5 p.m. every day after ONC for 6 days (Day 1–Day 6), and the eyes were collected at noon of Day 7. Group 2: M109S (5 mg/kg; Red) was administered at 9 a.m. (Day 2-Day 7) and 5 p.m. (Day 1–Day 6) every day after ONC, and the eyes were collected at noon of Day 7. (**B**) The changes of body weights of mice treated with vehicle (bCD (15% beta-cyclodextrin)), 7.5 mg/kg M109S once a day, and 5 mg/kg M109S twice a day (total 10 mg/kg per day).

M109S was synthesized by Wuxi AppTech, as previously reported [12]. M109S was dissolved in 15% beta-cyclodextrin (15%-bCD, vehicle) at 2 mg/mL concentrations and was administered orally to the mice through oral gavage. A total of 44 mice were randomly assigned to three groups, vehicle (15%-bCD), 7.5 mg/kg M109S once a day or 5 mg/kg M109S twice a day (total 10 mg/kg per day). Mice were not subjected to food restrictions and had 24-h access to food and water. In the 1st group, M109S (7.5 mg/kg) (2 mg/mL) or vehicle was administered at 5:00 p.m., starting from the day of ONC surgery, and continued for one week. In the 2nd group, M109S (5 mg/kg twice a day) (2 mg/mL) was administered orally at 9:00 a.m. and 5:00 p.m. (See Figure 1 for time course schematic).

### 2.4. Immunohistochemistry (IHC)

**Figure 2:**
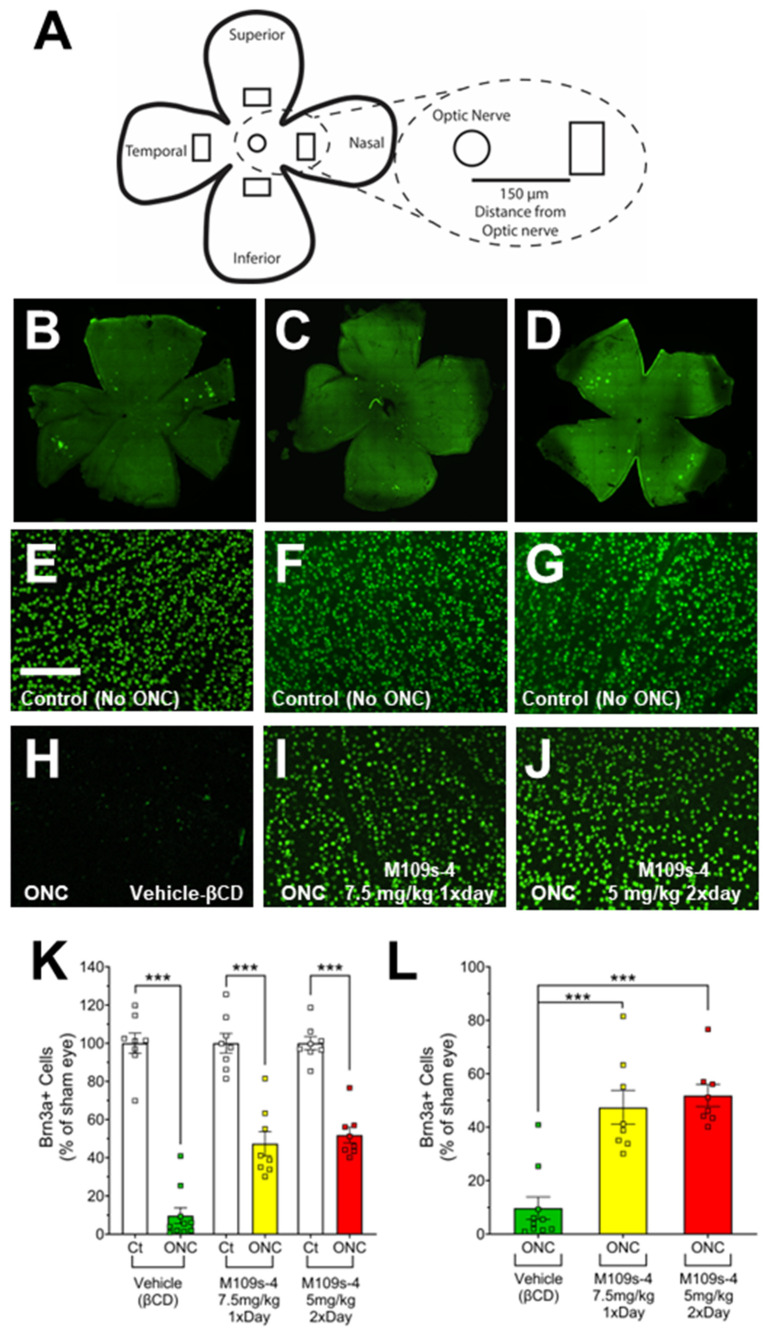
M109S attenuated the decrease of BRN3A-positive (BRN3A^+)^ RGCs in ONC-treated retina. ONC and M109S administration procedures were conducted according to the protocol outlined in Figure 1A. BRN3A^+^ cell counts were performed on retinas from both sham and ONC-treated eyes. (**A**) Schematic of whole retina flat mount showing an approximate location where images were taken. The circles denote the optic nerve, and the rectangles represent the location where images were taken. (**B**–**D**) Tiled images of a retina stained with anti-Brn3a and the Alexa-488 (green fluorescence)-conjugated secondary antibody; (**E**–**G)** Immunostaining of BRN3A in the retina from sham eyes; (**H**–**J**) Immunostaining of BRN3Ain the retina of the ONC-treated eyes. (**B**,**E**,**H**) Vehicle-treated control mice; (**C**,**F**,**I**) 7.5 mg/kg M109S once a day; (**D**,**G**,**J**) 5 mg/kg M109S twice a day (total 10 mg/kg per day). (**K**) The data are shown as the percentages of the number of BRN3A^+^ cell relative to sham eyes, with the average number of Brn3a^+^ cells in sham eyes designated as 100% for each treatment group. (**L**) The graph showed only the ONC-treated retina (The results of sham control are removed from K). ONC treatment induced a drastic decrease of BRN3A+ cells in vehicle control mice. On the other hand, mice treated with M109S at 7.5 mg/kg (once a day) or 5.0 mg/kg (twice a day) exhibited a substantial rescue in Brn3a^+^ RGCs. The data are presented as the percentage of BRN3A^+^ cell survival ± SEM for vehicle control (No-ONC (n = 8) and ONC (n = 10)), M109S 7.5 mg/kg (once a day) (n = 8), and M109S 5.0 mg/kg (twice a day) (n = 8). Statistical significance is denoted as *** *p* < 0.001. Scale bar = 150 μm.

BRN3A (brain-specific homeobox/POU domain protein 3A) was used as an RGC marker [15], and cleaved caspase-3 was used as an apoptotic cell marker [16]. Eyeballs were collected three or seven days after ONC. The eyes were removed, and the cornea and lens were removed. The retinas were fixed in 4% paraformaldehyde (PFA) (dissolved in phosphate buffer saline (PBS)) for 1 h at room temperature. The retinas were then permeabilized with 1% Triton X-100 in PBS at room temperature and washed three times with PBS. Floating retinas were incubated with rabbit mono-clonal-antiBRN3A antibody (ab245230, Abcam, Waltham, MA, USA) diluted 1:100, mouse mono-clonal-anti-BRN3A antibody conjugated to Alexa 594 (sc-8429 AF594, Santa Cruz Biotechnology, Dallas, TX, USA) diluted 1:100, or cleaved caspase-3 antibody (9664S, Cell Signaling Technology, Danvers, MA, USA) diluted 1:500 in blocking buffer (PBS, 0.1% bovine serum albumin (BSA), 1% Triton) for four days at 4 °C. After four days, the retinas were washed three times in PBS and incubated for 2 h at room temperature with the secondary antibody (Alexa 488, diluted 1:500 in blocking buffer (PBS, 0.1% BSA)). The retinas were then mounted on slides and imaged using fluorescence microscopy (EVOS M7000). The data analysis involved examining mice across all experimental conditions. In total, 8–10 mice were included in each cohort. For each mouse, images were quantified to analyze the relevant parameters. These images were taken approximately 150 µm from the optic nerve (Figure 2A), providing a consistent reference point for analysis. Specifically, at least 2–4 images were averaged for each retina to ensure representative data collection. This approach helped mitigate potential variability and provided a robust dataset for analysis. 

### 2.5. Image Analysis

Image analysis was performed using ImageJ software (version 1.54d). Briefly, the images were opened in ImageJ and converted to 8-bit grayscale. A threshold was applied to the images to segment the BRN3A-positive (BRN3A^+^) or cleaved caspase-3-positive cells from the background. The “Analyze Particles” function was used to count the number of BRN3A^+^ cells in each image. The number of BRN3A^+^ or cleaved caspase-3-positive cells was com-pared to the total number of BRN3A^+^ cells from their control retina. 

### 2.6. Histological Analysis of the Lungs and Kidney

The lung and kidney were collected from the mice treated with vehicle or M109S (5 mg/kg twice a day) for 7 days. As the positive control of the abnormal kidney histology, we used Cisplatin, which is known to induce acute kidney injury (AKI) [17]. To induce acute kidney injury (AKI), mice were treated with 20 mg/kg of cisplatin dissolved in saline as previously reported [17]. Three days after Cisplatin treatment, the kidneys were collected for histological analysis. The kidneys were fixed by 4% PFA and the paraffin sections (4 um) were stained with Hematoxylin/Eosin (HE). Histological slides of kidney tissue were assessed blindly using a semiquantitative scoring system to gauge acute kidney injury (AKI) as previously described [18]. In this method, 100 cortical tubules from 3 fields were randomly selected and scored as described previously [18]. As the positive control of the abnormal lung histology, the lung sections from Ku70 knock-out (KO) mice were used, which display enlargement of alveolar space due to the excess BAX-induced lung alveolar cell death [19,20]. Lungs were collected and fixed by intratracheal instillation of 4% paraformaldehyde to maintain the alveolar structure according to previously published methods [19,20]. Tissue sections (7 μm thick) from the upper left lobe were prepared for analysis. Mean linear intercept measurements were employed to assess differences in alveolar size, following previously established methods as previously reported [19,20]. At least 12 representative fields of lung sections were photographed, excluding large airways and blood vessels.

### 2.7. Data Analysis

Data were analyzed using a one- or two-way analysis of variance (ANOVA) (GraphPad Prism 10.1, GraphPad Software Inc., San Diego, CA, USA) followed by a Tukey post-hoc test. Results were considered statistically significant at *p* < 0.05.

## 3. Results

### 3.1. The Time Schedule of ONC and M109S Treatment

As shown in Figure 1A, ONC was performed at noon on Day 1. At 5 p.m. of Day 1, the oral administration of M109S was started with two different doses, as explained in detail in the Materials and Methods. M109S was administered once (7.5 mg/kg) or twice (5 mg/kg) a day for 6 days or 7 days, respectively. The eyes were collected by noon on Day 7 (10 a.m.–12 p.m.). Figure 1B shows the body weight of the mice treated with ONC and M109S or vehicle control. The mean body weight values for the M109S-treated group did not show any remarkable deviations from the control and other experimental groups during the observation period. ANOVA results indicated no significant difference in body weight between all groups at 7 days post-ONC (F_(3, 31)_ = 0.21; *p* = 0.89). 

### 3.2. M109S Attenuated the Decrease of BRN3A-Positive (BRN3A^+^) Cells in the Retinal of ONC-Treated Mice

The effects of M109S on RGC were assessed by the immunohistochemical staining of BRN3A from the retina collected on Day 7 (Figure 2). For each mouse, images were taken approximately 150 µm from the optic nerve (Figure 2A). Retinas from sham eyes that did not receive ONC showed robust staining of BRN3A^+^ cells in vehicle-treated (Figure 2E), 7.5 mg/kg M109S once a day (Figure 2F), and 5 mg/kg M109S twice a day (total 10 mg/kg per day) (Figure 2G). The retinas subjected to ONC and treated with vehicle showed a significant decrease of BRN3A^+^ cells (9.75% ± 4.14 of sham control, n = 10) (Figure 2H,K,L). In contrast, mice treated with 7.5 mg/kg M109S once a day, post-ONC, showed a robust preservation of BRN3A^+^ RGCs (47.35% ± 6.30 of sham control, n = 8) (Figure 2I,K,L). Treatment with 5 mg/kg M109S twice a day (total 10 mg/kg per day), post-ONC, also showed a robust preservation of BRN3A^+^ RGCs (51.83% ± 4.14 of sham control, n = 8) (Figure 2J–L). The statistical analysis using a one-way ANOVA demonstrated a significant difference between all groups (F_(5, 44)_ = 66.74; *p* < 0.001). Post hoc analysis (Tukey post hoc test) indicated a significant rescue of BRN3A^+^ cells in all M109S treated retinas compared to vehicle-treated retinas (Figure 2K,L). 

### 3.3. M109S Inhibited ONC-Induced Caspase-3 Activation in the Retina

**Figure 3:**
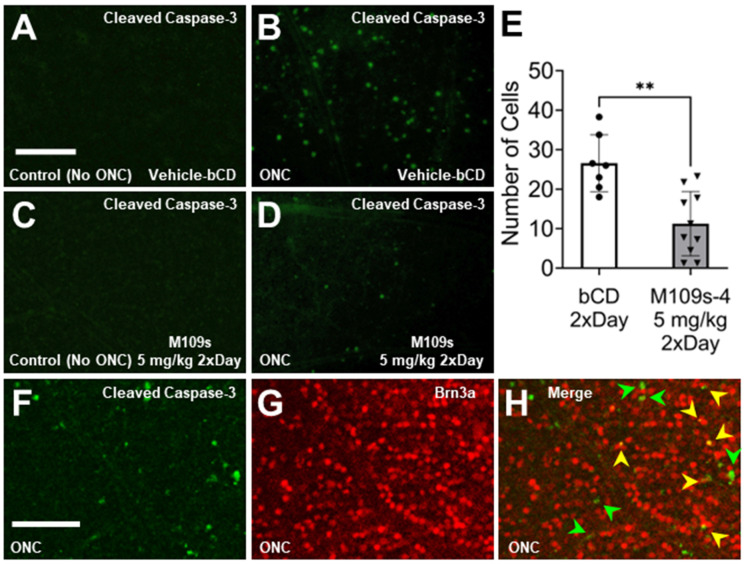
M109 treatment suppressed ONC-induced Caspase-3 activation in the retina. (**A**–**D**) The retina was stained with anti-cleaved Capase-3 (activated Caspase-3) and Alexa-488-conjugated secondary antibody. Caspase-3-positive cells were detected in the retina collected on Day 3 after ONC (**B**,**D**) but not in the control retina (**A**,**C**). In the ONC-treated eyes, M109S treatment (5 mg/kg M109S twice a day decreased the number of active-Caspase-3-positive cells in the retina of the ONC-treated eyes (**B**). To be noted, the staining of active Caspase-3 was detected in the cell body in the retina. (**E**) The quantitative analysis shows that the effects of M109S to decrease the number of active Caspase-3-positive cells were statistically significant (*p* = 0.0012). Circles and triangles denote individual retinas treated with Vehicle and M109S (administered at 5 mg/kg twice daily), respectively. (**F**,**G**) The retina collected 3 days after ONC was stained with antibodies detecting cleaved (activated) Caspase-3 (green) and Brn3a (red). (**H**) Merged image of F and G detecting cleaved (activated) Caspase-3 (green) and Brn3a (red). There are cells expressing active Caspase-3 (green arrows) and cells expressing both active Capase-3 and BRN3A (yellow arrows) indicating that some of the BRN3A^+^ RGCs undergo apoptosis within 3 days after ONC. The data are presented as the number of Caspase-3-positive cells ± SEM for vehicle control (n = 7), and M109S 5.0 mg/kg (twice a day) (n = 10). A total of four images were averaged for each retina to ensure representative data collection. Statistical significance is denoted as ** *p* < 0.01. Scale Bar = 150 μm.

**Figure 4:**
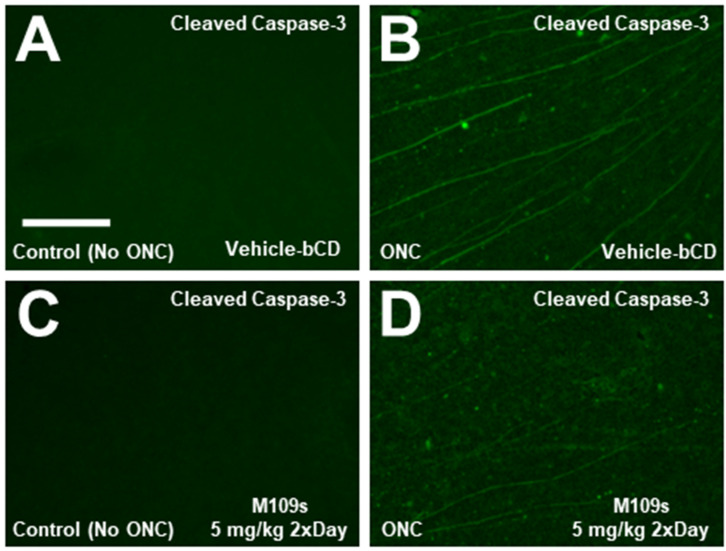
Active Caspase-3 was detected mainly in the axon of the retina of the ONC-treated eyes collected on Day 7. (**A**–**D**) The retina was collected on Day 7 after sham (**A**,**C**) or ONC (**B**,**D**) and stained with anti-cleaved Capase-3 (activated Caspase-3) and Alexa-488-conjugated secondary antibody. Active Caspase-3 was detected only in the ONC-treated retina. Unlike the retina collected on Day 3 after ONC (Figure 3), active Capsase-3 staining was detected mainly in the axon but not in the cell body. The staining of active Capsase-3 seems to be decreased by M109S treatment (5 mg/kg twice a day), however, the quantitative analysis of the number of active Caspase-3-positive cells was not possible since the axons were mainly stained with anti-active Caspase-3, as explained in the main text. Scale bar = 150 μm.

To determine the effects of M109S on apoptosis induction, the retina was stained with anti-active Caspase-3 (cleaved Caspase-3). On Day 3, after ONC, active Caspase-3 was detected in the cell bodies of ONC-treated retinas, whereas almost no active Caspase-3 was detected in the sham control (Figure 3A,C). The number of cells with active Capase-3 was counted in each retina, and the effects of M109S on the appearance of active Caspase-3-positive cells were assessed. As shown in Figure 3D,E, M109S treatment (5 mg/kg twice a day) inhibited the occurrence of Caspase-3 activation (t_(15)_ = 3.99, *p* = 0.0012). Since the number of BRN3A^+^ RGCs decreased by ONC, we examined whether we could capture BRN3A^+^ RGC undergoing apoptosis by the double staining for BRN3A and cleaved (active) Caspase-3. As shown in Figure 3F–H, some of the Brn3a^+^ RGCs were double stained with anti-active Caspase-3 (these cells are indicated by yellow arrows in Figure 3H), indicating that some of BRN3A^+^ RGCs undergo apoptosis within 3 days after ONC. BRN3A is known to be expressed in the nucleus [15], however, Caspase-3 is known to be activated in the cytosol, and the activated Caspase-3 distribute to the nucleus at a later stage of apoptosis [10,16]. Therefore, some of the BRN3A^+^ (nuclear staining) cells that have close staining of active Caspase-3 (it may be the cytosolic Caspase-3 staining) may be also undergoing apoptosis (these cells are indicated with green arrows in Figure 3H). We also stained the retinas collected on Day 7 after ONC treatment. Interestingly, active Caspase-3 was detected in the axon rather than the cell body of the retina collected at Day 7 (Figure 4). Since active Caspase-3 staining was detected mainly in the axon of the retinas collected on Day 7, it was not possible to count the number of active Caspase-3-positive cells to determine the effects of M109S on this day. Therefore, we determined the effects of M109S on Caspase-3 activation using the retina collected on Day 3 as explained above (Figure 3). 

### 3.4. M109S Did Not Induce Abnormal Histology in the Lungs and Kidneys

**Figure 5:**
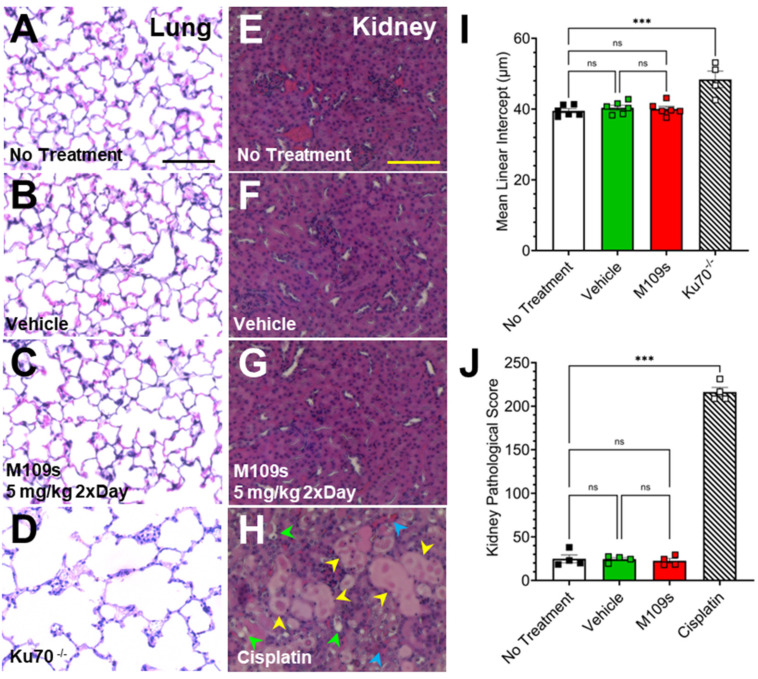
M109S did not cause detectable toxicities in the lung and kidney after seven continuous days of treatment in mice. The hematoxylin and eosin (HE) staining of the paraffin sections of (**A**–**D**) the lung and (**E**–**H**) kidney are shown. (**A**–**C**,**E**–**G**) The lung and kidney were collected after 7 days of continuous treatment of vehicle control or M109S (5 mg/kg twice a day). The lung tissue of Ku70 KO mouse (**D**) and the kidney tissue of cisplatin-treated mice (**H**) are shown as examples of the organ damages. (**I**) Lung tissue from Ku70^-/-^ mice showed significantly higher values of mean linear intercept in comparison with other three groups (no treatment, vehicle-, and M109S-treated mice). M109S treatment did not induce significant changes of the lung alveolar size in comparison with control groups. (**J**) Cisplatin-treated mice showed a significantly higher kidney damage score in comparison with other three groups (no treatment, vehicle and M109S treated mice). M109S treatment did not induce significant changes of the kidney pathological score in comparison with control groups. Lung data are presented as the mean ± SEM for no treatment (n = 6), vehicle control (n = 6), M109S 5.0 mg/kg (twice a day) (n = 6) and Ku70 KO (n = 4). Kidney data are presented as the mean ± SEM for no treatment (n = 4), vehicle control (n = 4), M109S 5.0 mg/kg (twice a day) (n = 4), and cisplatin-treated (n = 4). Yellow arrows indicate tubular epithelial cell flatting; green arrows indicate brush order loss; blue arrows indicate tubuler lumen obstruction. No significant difference is indicated with “ns”. Statistical significance is denoted as *** *p* < 0.001. Scale bar = 75 μm.

After seven days of treatment of M109S (5 mg/kg twice a day, total of 10 mg/kg/day), the lungs and kidneys were collected, and the histology of these organs was examined (Figure 5). The organs were also collected from control mice (vehicle), cisplatin-treated mice, and non-treated mice. The kidney tissues from cisplatin-treated mice were used as an example of drug-induced histological damage to the kidney which involves BAX-mediated cell death [17]. It has been shown that the cisplatin-induced acute kidney damage is significantly ameliorated by BAX gene KO [17]. Data from the lung tissue collected from Ku70 KO (Ku70^-/-^) mice were previously reported by the authors [19] and used here as an example of damaged lung tissue. The lungs in Ku70 KO mice showed enlarged alveolar space (Figure 5D) due to the excess BAX-mediated lung alveolar epithelial cell death as previously reported [19]. This abnormal lung histology of Ku70 KO was corrected by BAX gene KO [19]. 

The lung alveolar size was quantitatively analyzed by a previously established method of calculating the mean linear intercept, an indicator of alveolar size [19]. One-way ANOVA showed a significant difference of mean linear intercept values between Ku70 KO lung tissue and all other treatment groups (F_(3, 18)_ = 13, *p* < 0.001) (Figure 5I). Post hoc analysis (Tukey post hoc test) indicated significant damage in the Ku70 KO lung tissue compared to no treatment, vehicle-treated, and M109S-treated mouse lung tissue (Figure 5I). There were no statistically significant differences among no treatment, vehicle-, and M109S-treated groups, suggesting that M109S did not cause lung tissue damage (Figure 5A–D,I).

The kidney tissue from cisplatin-treated mice showed significant damage, as shown in Figure 5H. For example, the damage can be detected as tubular epithelial cell flatting, brush border loss, or tubular lumen obstruction. A previously established quantitative method scoring kidney damage (kidney pathological scoring method) was used to compare the effects of cisplatin and M109S. A one-way ANOVA showed a significant difference between cisplatin-treated mice and all other groups (F_(3, 12)_ = 625.2, *p* < 0.001) (Figure 5J). Post hoc analysis (Tukey post hoc test) indicated a significant damage in cisplatin-treated kidney tissue compared to no treatment, vehicle-treated, and M109S-treated mouse lung tissue (Figure 5J). M109S, as well as vehicle groups, did not show a significant increase of the kidney pathological score suggesting that M109S did not cause kidney toxicity (Figure 5E–H,J).

## 4. Discussion

The present study demonstrated that the orally administered M109S attenuated RGC degeneration induced by ONC in mice. In this study, the dose of 7.5 mg/kg (once a day) or 5 mg/kg (twice a day) showed statistically significant effects on inhibiting RGC degeneration. The protective effects were approximately 50% suppression of BRN3A^+^ RGC degeneration (Figure 2). We speculate that more protective effects of M109S can be achieved by improving the M109S administration strategy. Further studies are warranted to develop the most effective strategy (by optimizing dose, vehicle, and/or administration route) to achieve the maximum protection of RGCs from traumatic injuries.

Previous studies showed that apoptosis plays important roles in RGC death after ONC [16]. In fact, we observed that the activated Caspase-3 became detectable in the retina after ONC (Figure 3 and Figure 4). Interestingly, the staining patterns of activated Caspase-3 showed a time-dependent change. Three days after ONC, the cell body was stained and, therefore, we were able to count the number of apoptotic cells (Figure 3). Seven days after ONC, the staining was detected mainly in the axon, but not in the cell body (Figure 4). These results suggest that the signals of active Caspase-3 migrate from the cell body to the peripheral axons during the 7-day period after the ONC. This transition of active Caspase-3 in RGCs suggest that the early apoptotic event is initiated in the cell body. Since Caspase activation begins in the cell body localized on the surface of the retina, effective drug delivery to the retinal surface is necessary to prevent RGC loss after an optic nerve injury. The orally administered M109S reaches the retina by penetrating the blood–retina barrier [12]. In our previous study, we showed that approximately 40% of blood M109S entered the retina [12]. To improve the protection of retinal cells, the local and direct administration of M109S to the eye may be also effective. 

The present study showed that BRN3A+ cells expressed active Caspase-3, which is the hallmark of apoptotic events. M109S decreased the number of active Caspase-3 expressing cells indicating that M109S suppressed the occurrence of apoptosis in the retina of the mice treated by ONC. In addition to apoptosis inhibition, M109S may have unexpected impact on the gene expression of BRN3A. It is because previous study showed that the gene expression of BRN3A was decreased in the survived RGC in the retina of ONC-treated mice [15,21,22]. Therefore, the suppression of the decrease of BRN3A-positive cells may be partly due to the maintenance of BRN3A expression of the survived RGCs.

The aim of the present study was to prove the concept that the orally administered M109S can inhibit acute apoptosis of RGCs in the retina induced by optic nerve injury. We elected to use BRN3A as a marker for RGCs, as previous studies showed that a significant decrease (approximately 60% decrease of BRN3A^+^ cells) of BRN3A^+^ cells occur within one week after ONC [23]. There are other RGC makers such as beta-III Tubulin that has been used as a marker of RGC [24,25]. Previous studies showed that a significant decrease of the beta-III Tubulin-positive cells occurred 2–3 weeks after ONC [10,23] (approximately 30% after 2 weeks [23] or a 50% decrease after 3 weeks [10]). To obtain further evidence that M109S has a therapeutic effect to prevent blindness caused by optic nerve injury, additional studies will be necessary to determine whether a longer M109S treatment duration (2 weeks and longer) can inhibit the decrease of the numbers of beta-III Tubulin-positive cells. 

A recent study showed that the regeneration of the damaged RGC axon was promoted by a small compound called M1 that stimulates mitochondrial fusion and transport [26]. In this study, M1 was applied directly to the injured site (damaged axon) [26]. The mechanism of action of M1 is to promote axon regeneration by enhancing mitochondrial fusion and transport which is distinct from the mechanism of action of M109S. Therefore, a combinational treatment of M1 and M109S may result in an additional or a synergistic protection of the damaged RGC. Another recent study showed that adenovirus-mediated gene delivery of *Bcl-XL* into the retina was effective in suppressing RGC degeneration in a mouse glaucoma model [27]. *Bcl-XL* is an anti-apoptotic member of *Bcl-2* family proteins that inhibits BAX-mediated cell death [28]. The chronic expression of *Bcl-XL* by the gene delivery of *Bcl-XL* is an ideal strategy to prevent BAX-mediated cell death for a sustained period. The pharmacological inhibition of BAX by M109S is convenient, but daily oral administration is necessary to prevent BAX activation in the damaged cells. The oral administration of M109S will be an effective strategy to rescue RGC in acute conditions, however, the gene therapy-based strategy to inhibit BAX activation may have an advantage if the side effects of virus-mediated gene therapy are negligible. 

## 5. Conclusions

In summary, the present study showed the oral bioavailability and neuroprotective efficacy of M109S in a mouse model of acute optic nerve injury. Previously, we reported that M109S protected mouse photoreceptor cells from bright light-induced cell death in two different mouse strains (*Abc4^-/-^Rdh8^-/-^* and *Balbc/J*) [12]. BAX-mediated unwanted cell death is known to induce various degenerative diseases including Alzheimer’s disease [29], Parkinson disease [30], Amyotrophic Lateral Sclerosis [31,32,33], Huntington disease [34], ischemia-reperfusion tissue injuries [3,35,36], and glaucoma [3,10]. The present and previous studies demonstrating the efficacy of M109S in mouse retinal disease models suggest that M109S has a potential to become the basis of future orally available therapeutics protecting essential cells from unwanted BAX-mediated cell death in various diseases.

## 6. Patents

M109S is listed as one of the cytoprotective small compounds (CPSCs) developed by Shigemi Matsuyama in the US patent application published in 2020 (Matsuyama, S., Greenlee, W. (2020). The use of novel small compounds of BAX inhibitors to protect damaged cells from death. US Patent Office, https://patents.google.com/patent/WO2021002986A2/en) (accessed on 29 December 2023).

## Data Availability

The data of the present study is available on request to the corresponding author.
